# The Cambridge Structural Database (CSD): Current Activities and Future Plans

**DOI:** 10.6028/jres.101.024

**Published:** 1996

**Authors:** David G. Watson

**Affiliations:** Cambridge Crystallographic Data Centre, 12 Union Road, Cambridge CB2 1EZ, England

**Keywords:** chemical connectivity representations, database use, disorder, high polymers, nonbonded contact preferences, search software

## Abstract

This paper reviews the search and analysis software packages QUEST3D and VISTA, also the database-building program PreQuest. The relationship between the CSD and the Protein Data Bank is discussed and development plans are outlined.

## 1. Introduction

The Cambridge Structural Database (CSD) covers the crystal structures of organic and metal-organic compounds. A few statistics illustrate the current size and growth rate of the CSD. The database currently contains 146 272 entries corresponding to 137 268 structures. The difference between these two numbers reflects the fact that there are multiple studies for certain compounds. The number of entries archived during the past year is 11 076 and 54 % of these relate to metalorganic structures. The emphasis on the structure of metal-organic structures has been steadily increasing over the last few years.

The Cambridge Crystallographic Data Centre (CCDC) provides four software packages for use with the CSD. These are:
QUEST3DThe search programVISTAThe data analysis programGSTATA geometrical analysis program, now superseded by VISTAPLUTOA program for plotting crystal structures.

In this paper some of the major features of QUEST3D and VISTA will be described.

## 2. QUEST3D

Although named QUEST3D, in fact this program caters for 1D, 2D, and 3D searches.

### 2.1 1D Searching

This involves the searching of textual and numeric data. Examples of the former would be searches of compound names, authors’ names, and space group symbols. Numeric searches might involve reciprocal cell parameters, metric classes, space group numbers, publication years, etc. Additionally searches can be conducted on elemental composition and chemical formula. A special 1D search is the peptide sequence search. The CSD contains more than 1000 entries for peptides of 2–25 amino-acids. These can be searched in terms of acyclic/cyclic structures, internal sequences or terminal residues, simple or substituted amino-acids, exact or wild-card residues.

### 2.2 2D Searching

This is the search for 2D chemical structures or substructures. The chemical fragment of interest is constructed using a BUILD menu and 2D-CONSTRAIN sub-menu. BUILD allows the construction of the skeleton in terms of element and bond types whereas 2D-CONSTRAIN allows the user to impose constraints on the fragment. These might concern the numbers of terminal hydrogen atoms, total coordination numbers, acyclic or cyclic nature of bonds, etc. Judicious selection of the available constraints allows the definition of the chemical fragment to be as exact or as inexact as the user wishes.

In addition to the (sub)structure searching capability 2D similarity searching can also be performed. This type of search compares the chemical connectivity bit screens of the input structure against every compound in the database and calculates a similarity coefficient. The user specifies the number of hits, eg., 100, and the 100 most similar structures are saved.

### 2.3 Display of Hits

When a hit is registered the default display shows the 2D chemical diagram and the 3D crystal diagram in minimum overlap view. The user can select the 1D display for textual and numeric data pertaining to the hit and a number of features are available for examination of the 3D crystal structure. These include labelling by element type, coloring by element or bond type, calculation of specified interatomic distances and angles, also torsion angles. A dialbox is available which permits rotation, translation or scaling, either of the crystal chemical unit or the contents of the unit cell. Individual hits may be viewed and the hits kept or rejected; alternatively the search process can be switched to an automatic mode whereby all hits are kept until the end of the database has been reached.

### 2.4 3D Searching

Three-dimensional searching involves the combination of a 2D search for a chemical fragment coupled with 3D contraints describing the geometry of part or all of the fragment. The 3D constraints might be distances, angles or torsion angles between atoms in the search fragment, dummy atoms, planes, vectors, centroids. Very often in a first exploratory search pass the parameters are defined and tabulated for all search hits. In a second pass some of these parameters may be required to take values lying in certain ranges thereby limiting the hits from the search process.

An important difference between a 2D and 3D search is that, in the former, when a hit is registered the search moves on to the next entry in the database whereas in a 3D search the target structure is normally exhaustively searched to locate all occurrences of the fragment satisfying the 3D constraints.

Very importantly QUEST3D allows 3D searching both in terms of bonded and non-bonded interactions. In the case of non-bonded contact searches these can be conducted in terms of both intramolecular and intermolecular interactions, thereby allowing the user to study hydrogen bonding, pharmacophores and other novel interactions.

## 3. VISTA

VISTA is an elegant menu-driven program for the analysis of numeric data saved from a 3D search. The usual statistical functions can be calculated including histograms, scattergrams and principal component analysis. An important feature of the program is the ability to select a data point and view the corresponding chemical structure. This is very useful in attempting to account for outliers in a distribution. An array of editing features is also available allowing the user to add titles and select colors for the preparation of publication-quality diagrams and slides.

## 4. DBUSE

Over the last 10–15 years a number of scientists have used data contained in the CSD to conduct studies of the structures of important groups and analyze various structural systematics. The number of papers published as a result of these investigations is now about 500. Details of these have been compiled to constitute an ancillary database, DBUSE, which is searchable with the 1D keys of QUEST3D. In addition to the bibliographic details each entry contains a brief abstract of the published paper. An example of an entry in DBUSE is:

“Anomeric Orbital and Steric Control in Static Conformations and Systems Dynamics: Rotations of Methoxy Groups in 2,2 Dimethoxypropane and Similar Crystallographic COCOC Fragments

A. Cosse-Barbi, J.-E. Dubois

J. Am. Chem. Soc. 109, 1503–1511,1987

An analysis of 546 COCOC fragments retrieved from CSD is associated with MO calculations on dimethoxypropane, conformational INDO map and MO optimization of structural parameters. The conformations of cyclic and acyclic COCOC fragments have been examined and related to the anomeric effect. The structure correlation method is used to examine interconversion pathways.”

## 5. PDB

Various bibliographic records of entries in the Protein Data Bank have been incorporated into the CSD system and are searchable by the 1D keys of QUEST3D. Additionally the SEQRES records are searchable in a manner analogous to that used for small peptides mentioned in Sec. 2.1. An Xwindows link to the display program Rasmol has been provided so that the three-dimensional structure of the macromolecules can be studied and any sequence specified in the search process is highlighted in the 3D structure display.

## 6. PreQuest

Under development is a program called PreQuest which will be the principal CCDC database-building software tool. Importantly this package will be made available to users of the CSD system so that they can add their own structures, in CSD format, to their copy of the CSD. This facility has obvious applications, for example in a pharmaceutical company where in-house structures can be added long before they might be put in the public domain.

## 7. Current Developments

A number of projects are currently under development at the CCDC. These include:

- the input of high polymer data, eg. nylon, polyethylene etc., to the CSD
- the proper handling of disorder in terms of major and minor site occupancies
- collaboration with PDB staff on the connectivity representations of HET groups
- the provision of interfaces to modeling software packages
- the compilation of “standard” bond lengths for compounds involving s-block elements
- the rationalization and standardization of chemical connectivity representations
- the compilation of a library of nonbonded contact preferences.

## 8. Future Plans

A number of key projects have been identified for future activities. These include:

- the rationalization of multiple-study entries for the same chemical compound
- the re-design of data storage in the CSD to permit the inclusion of new data types
- the rationalization of QUEST/VISTA in terms of
  graphical query generator
  search engine
  browse and display module
  data analysis module.

